# Effectiveness of Empagliflozin-Linagliptin Fixed-Dose Combination on Chronic Kidney Disease Outcomes in Patients With Type 2 Diabetes in a Real-World Setting

**DOI:** 10.7759/cureus.100108

**Published:** 2025-12-26

**Authors:** Debmalya Sanyal, Soumyabrata RoyChaudhuri

**Affiliations:** 1 School of Medicine and Public Health, The University of Newcastle, Newcastle, AUS; 2 Endocrinology, Narayana Health Rabindranath Tagore International Institute of Cardiac Sciences (NH RTIICS), Kolkata, IND; 3 Endocrinology, Kali Prasad Chowdhury (KPC) Medical College and Hospital, Kolkata, IND

**Keywords:** chronic kidney disease (ckd), efficacy, empagliflozin, fixed-dose combination, linagliptin, type 2 diabetes mellitus

## Abstract

Introduction: Optimal management of chronic kidney disease (CKD) is essential in patients with type 2 diabetes mellitus (T2DM). Certain antihyperglycemic agents, such as empagliflozin, provide additional kidney protection beyond glycemic control, while linagliptin is renal-safe and associated with reductions in albuminuria. This retrospective study aimed to evaluate the long-term outcomes (at least one year) for CKD and T2DM with the innovator fixed-dose combination (FDC) of empagliflozin and linagliptin in sodium-glucose cotransporter-2 inhibitor (SGLT2-i)-naïve T2DM subjects who were previously uncontrolled on a dipeptidyl-peptidase-4 inhibitor (DPP4-i)-based regimen.

Methods: The study included case record analyses of T2DM patients followed in the outpatient setting for at least 12 months. Glycemic control and renal parameters, including estimated glomerular filtration rate (eGFR), eGFR slope, and changes in urine albumin-creatinine ratio (UACR), were evaluated.

Results: A total of 433 eligible case records were analyzed. Of the study participants, 63.3% were male, 54.1% had hypertension (HTN), and 10.3% had established atherosclerotic cardiovascular disease (ASCVD). A significant reduction in glycated hemoglobin (HbA1c) of 1.5% was observed from a baseline mean of 8.3 ± 1.7%, along with a mean body-weight reduction of 3.3 kg over 12 months of combination therapy. At baseline, the mean eGFR was 82.0 mL/min/1.73 m², with 20% of patients having an eGFR < 60 mL/min/1.73 m². The mean UACR was 207.8 mg/g, and 79% of patients had UACR ≥ 30 mg/g. An initial dip of 5.5% in mean eGFR (-4.5 mL/min/1.73 m²) was noted at three months following SGLT2-i initiation. After this early dip, the eGFR slope showed an upward trajectory and was 1.9 mL/min/1.73 m² above baseline at 12 months. A significant reduction in mean UACR of 142.9 mg/g was seen, decreasing from 207.8 mg/g at baseline to 64.9 mg/g at 12 months. At least a 30% reduction in UACR from baseline was achieved by 71.8% of patients. The odds of patients being in the A1 UACR category were 2.2-fold higher at 12 months.

Conclusion: In this observational study, the use of FDC of empagliflozin and linagliptin for at least 12 months in T2DM patients previously uncontrolled on a DPP4-i-based regimen was associated with reductions in albuminuria and improvement in eGFR slope after the expected initial dip at three months. Improvements in glycemic control and body weight were also observed, regardless of underlying cardiometabolic risk. The potential renal benefits noted in this descriptive evidence warrant further evaluation.

## Introduction

Type 2 diabetes mellitus (T2DM) is a major public health concern globally, with rising prevalence and significant morbidity and mortality. The Asian-Indian population is known for an early onset of T2DM and a high risk of vascular complications [1]. In specialized diabetes-care settings in India, at least one in three individuals with T2DM is estimated to have chronic kidney disease (CKD) [2]. Early CKD may not cause obvious symptoms, but kidney function can deteriorate rapidly as diabetic kidney disease progresses [3].

Optimizing the management of early CKD is therefore a key priority in T2DM. Recent studies provide evidence that certain antidiabetic agents offer kidney protection beyond glycemic control. In a real-world setting, there remains a need to identify treatment regimens that can effectively improve CKD outcomes in patients with T2DM. Early detection of diabetic kidney disease and preservation of renal function remain clinical challenges despite the use of renin-angiotensin-aldosterone system (RAAS) inhibitors. Achieving and maintaining glycemic control is known to delay the onset and progression of diabetic microvascular complications [4]. Managing T2DM involves not only maintaining glycemic control but also preventing and treating comorbidities and complications such as cardiovascular disease (CVD) and CKD [5]. Timely therapy adjustments and proactive management are crucial for improving outcomes in individuals with diabetes.

Urine albumin-creatinine ratio (UACR) of 30-299 mg/g represents moderately increased albuminuria (microalbuminuria) [6]. A consistent rise in UACR to this range indicates a higher risk of cardiorenal events and signals the need for early kidney-protective interventions. Screening for microalbuminuria in at-risk individuals enables early detection and timely measures to slow the progression of kidney disease [6,7]. A persistent increase in UACR to ≥300 mg/g indicates severely increased albuminuria (macroalbuminuria), which is associated with an even greater risk of cardiorenal events [6,8].

Glomerular filtration rate (GFR), measured using exogenous filtration markers, provides an accurate assessment of kidney function, but measuring true GFR in routine clinical practice is difficult. Estimated GFR (eGFR) remains the most practical and reliable tool for evaluating kidney function in both health and disease. Current recommendations favor the CKD-Epidemiology Collaboration (CKD-EPI) equation for assessing eGFR [6,7]. An eGFR consistently below 60 mL/min/1.73 m² indicates CKD and is linked with an increased risk of cardiorenal events, regardless of the patient’s albuminuria category [6,8].

It is well recognized that sustained albuminuria and sustained eGFR decline are independent risk factors for CKD progression, cardiorenal events, and mortality. A considerable proportion of patients with T2DM may show abnormalities in either UACR or eGFR, but not necessarily both. Therefore, it is essential to measure both eGFR and UACR at diagnosis and at least annually during follow-up, based on the patient’s prognostic risk status [6,7].

Evidence-based guidelines recommend personalized management of people with T2DM, guided by prioritized clinical goals and comorbidities [7,9]. Guidelines also suggest that initial combination therapy should be considered for patients presenting with HbA1c levels 1.5%-2.0% above target [5,9]. Early initiation of combination therapy has been proposed as a strategy to delay deterioration of glycemic control and potentially preserve β-cell function earlier in the disease course [10]. The fixed-dose combination (FDC) of the SGLT2-i empagliflozin and the DPP4-i linagliptin was the first such combination introduced in India in 2017 [11]. The empagliflozin-linagliptin FDC has been reported to provide effective glycemic control, early achievement of target HbA1c, and additional clinical benefits such as weight loss and blood pressure reduction [11]. The combination has also been hypothesized to carry a lower risk of genital mycotic infections compared with SGLT2-i monotherapy [12].

The Empagliflozin Cardiovascular Outcome Event Trial in Type 2 Diabetes Mellitus Patients-Removing Excess Glucose (EMPA-REG OUTCOME) study was the first to demonstrate a reduced risk of incident or worsening nephropathy with sodium-glucose cotransporter-2 inhibitor (SGLT2-i) inhibition. These benefits were driven by a slower decline in eGFR and a reduction in albuminuria in patients with T2DM and established CVD [13]. In this trial, empagliflozin was also associated with significantly lower odds of rapid eGFR decline [14]. Following this, several major SGLT2-i trials have consistently shown reduced risk of CKD progression to kidney failure in patients with or without underlying T2DM [15]. A meta-analysis of cardiovascular outcome trials by McGuire et al. reported a 38% relative risk reduction in kidney outcomes with SGLT2-i inhibition among patients with T2DM at elevated cardiovascular risk, regardless of underlying ASCVD status [16].

Linagliptin, a DPP4-i approved for glycemic control in T2DM, does not require dose adjustment in individuals with CKD due to its predominantly biliary elimination [17]. The Cardiovascular and Renal Microvascular Outcome Study with Linagliptin (CARMELINA) provided conclusive evidence of both cardiovascular and renal safety in high-risk patients with T2DM, CKD, and CVD [18,19]. In this trial, linagliptin use was also associated with slower progression and greater regression of albuminuria [18,19].

Management of CKD in patients with T2DM involves addressing multiple risk factors and incorporating kidney-protective therapies [6,7]. At the same time, a simplified and personalized treatment approach is needed to help patients meet various clinical goals while maintaining adherence. The original FDC of empagliflozin and linagliptin offers consistent glucose-metabolic control with fewer pills, minimal dosing considerations, broad patient eligibility, and a well-established safety profile, along with supportive evidence in CKD [11,19].

In this context, the primary objective of this study is to evaluate changes in albuminuria and renal function over 12 months of treatment with the original empagliflozin-linagliptin FDC in patients with T2DM who were previously uncontrolled on a DPP4-i regimen and were SGLT2-i-naïve. Secondary objectives include assessing changes in glycemic control, body weight, and blood pressure. This study is based on case record analyses from two tertiary diabetes-care centers in eastern India.

## Materials and methods

Study design and population

This real-world retrospective study was conducted on a cohort of patients with type 2 diabetes mellitus (T2DM) receiving routine care at two tertiary-care clinic settings in eastern India from 2021 to 2024. Inclusion criteria were adults aged 18 years or older with a diagnosis of T2DM who were newly initiated on empagliflozin-linagliptin FDC after inadequate control on a DPP4-i-based antidiabetic combination therapy (with no prior exposure to SGLT2-i). Patients on angiotensin-converting enzyme inhibitors (ACE-i) or angiotensin receptor blockers (ARBs) as antihypertensives, and who received calcium channel blockers (CCBs) as the first add-on for hypertension (HTN) at maximally tolerated doses, were included. Case records were eligible only if the patient continued empagliflozin-linagliptin FDC for at least 12 months and had follow-up details documented at the recommended visits during this period.

Exclusion criteria included patients requiring rescue therapy with glucagon-like peptide-1 receptor agonists (GLP-1 RAs) or insulin, as well as those initiated on non-steroidal mineralocorticoid receptor antagonists (ns-MRA, e.g., finerenone). Patients receiving antihypertensives other than ACE-i/ARB and CCB at baseline or as rescue therapy were excluded. Those with any prior SGLT2-i exposure, either as monotherapy or in combination, were also excluded to avoid confounding in renal outcome assessment.

Only patients with complete data for all clinical variables at baseline and at 12 months were included in the respective analyses.

Data collection

Data were collected retrospectively from electronic health records (EHRs) of the two tertiary-care centers. All case records available from January 2021 to December 2024 (both months included) that met the eligibility criteria were screened. From these, only cases with complete information for the primary outcome measures (UACR and eGFR at baseline and 12 months) were included in the analysis. The collected data included demographic details, clinical parameters, and laboratory results. Baseline characteristics recorded were age, gender, duration of T2DM, HbA1c levels, and any history of atherosclerotic cardiovascular disease (ASCVD), heart failure (HF), or HTN.

Laboratory parameters

HbA1c was measured using high-performance liquid chromatography (HPLC) on the Variant II Turbo-D100 system (Bio-Rad Laboratories). UACR was assessed using immunoturbidimetric assays, with urine albumin measured by immunoturbidimetry and urine creatinine by the Jaffe reaction on Roche Cobas 8000 analyzers. UACR values were categorized into A1 (<30 mg/g), A2 (30-299 mg/g), and A3 (≥300 mg/g). eGFR was calculated using the CKD-EPI 2021 equation.

Outcome measures

The primary outcome measures were the changes in UACR and eGFR over 12 months. A 30% reduction in UACR and stabilization of the annual eGFR slope were considered clinically meaningful. The annual eGFR slope was assessed using records with complete eGFR data at baseline, three months, six months, and 12 months. Secondary outcomes included the relationship between glycemic control (HbA1c) and other metabolic parameters.

Statistical analysis

Descriptive statistics were used to summarize the baseline and 12-month characteristics of the study population. Continuous variables were expressed as mean ± standard deviation (SD), and categorical variables as frequencies and percentages. Paired t-tests were used to compare baseline and 12-month values of UACR and eGFR. Fisher’s exact test was used to identify factors associated with achieving at least a 30% reduction in UACR. Repeated-measures ANOVA was performed to assess time-based changes in eGFR in the eGFR slope analysis. A p-value < 0.05 was considered statistically significant.

For estimating the required sample size, clinically meaningful benefits were defined as achieving at least a 30% reduction in UACR from baseline to 12 months, and stabilization of eGFR with <1 mL/min/1.73 m² decline from the 3rd to the 12th month. Assuming that at least 50% of patients would achieve these benefits, with a 95% confidence level and 5% margin of error, a minimum of 385 patients was required for meaningful statistical analysis. All statistical tests were performed using Jamovi Cloud version 2.7.6.

## Results

**Figure 1:**
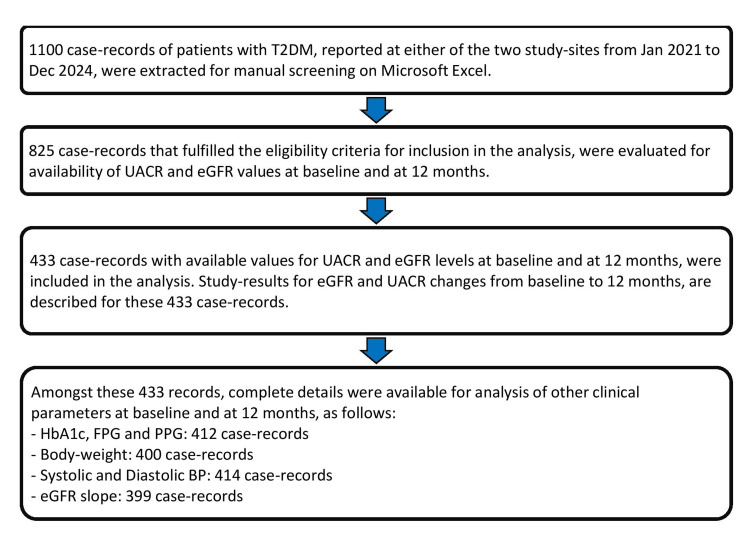
Flow diagram of case record selection for analysis

The study population included case records of 433 patients. The flow diagram outlining the case record selection process for the analysis is shown in Figure 1.

At baseline, the mean age of the study population was 60.3 years, with 63.3% being male, and a mean duration of T2DM of 6.6 years. The mean UACR level was 207.8 mg/g, with 79% of patients having UACR ≥ 30 mg/g. The mean eGFR was 82.0 mL/min/1.73 m², and 20% of patients had eGFR < 60 mL/min/1.73 m². Baseline clinical characteristics of the study population are summarized in Table 1.

**Table 1:** Baseline demographics of the study population T2DM: type 2 diabetes mellitus, ASCVD: atherosclerotic cardiovascular disease, UACR: urine albumin-creatinine ratio, eGFR: estimated glomerular filtration rate.

Demographic variables	Total study population (N = 433)
Age (years), mean (SD)	60.3 (10.5)
Male gender, n (%)	274 (63.3)
Duration of T2DM (years), mean (SD)	6.6 (5.5)
Patients with history of ASCVD, n (%)	44 (10.3)
Patients with history of heart failure, n (%)	118 (27.6)
Patients with history of hypertension, n (%)	231 (54.1)
UACR (mg/g), mean (SD)	207.8 (426.8)
eGFR (mL/min/1.73m^2^), mean (SD)	82.0 (23.1)

The clinical parameters, HbA1c, fasting plasma glucose (FPG), and post-prandial plasma glucose (PPG), showed statistically significant reductions from baseline. Changes in these glycemic parameters over 12 months of therapy, analyzed in 412 patients, are summarized in Table 2.

**Table 2:** Clinical characteristics of HbA1c, FPG, and PPG: difference from baseline to 12 months HbA1C: glycated hemoglobin, FPG: fasting plasma glucose, PPG: post-prandial plasma glucose.

Clinical characteristics (N = 412)	Baseline, mean (SD)	12 months, mean (SD)	Difference, mean (SD)	Test statistic, p-value
HbA1c (%)	8.3 (1.7)	6.7 (0.4)	-1.5 (1.59)	t -19.74, p < 0.001
FPG (mg/g)	163.6 (62.4)	114.6 (12.3)	-49.1 (63.2)	t -15.76, p < 0.001
PPG (mg/g)	243.5 (92.5)	155.1 (21.5)	-88.3 (91.8)	t -18.95, p < 0.001

Changes in body weight over 12 months were evaluated in 400 patients. The mean (SD) body weight decreased from 69.7 (11.1) kg at baseline to 66.4 (10.3) kg at 12 months, representing a mean (SD) change of -3.3 (7.2) kg (t = -9.11, p < 0.001).

Reduction in systolic blood pressure (SBP) and diastolic blood pressure (DBP) was not statistically significant in the overall cohort of 414 patients (Table 3). However, in a subset of 252 patients with baseline SBP ≥ 130 mmHg, mean SBP decreased by -8.9 mmHg (p < 0.05), and in 217 patients with baseline DBP ≥ 80 mmHg, mean DBP decreased by -5.5 mmHg (p < 0.05).

**Table 3:** Clinical characteristics of SBP and DBP: difference from baseline to 12 months SBP: systolic blood pressure, DBP: diastolic blood pressure.

Clinical characteristics	Baseline, mean (SD)	12 months, mean (SD)	Difference, mean (SD)	Test statistic, p-value
Weight (kg) (N = 400)	69.7 (11.1)	66.4 (10.3)	-3.3 (7.2)	t -9.11, p < 0.001
SBP (mmHg) (N = 414)	132.5 (16.5)	131.8 (10.7)	-0.7 (15.5)	t -0.95, p = 0.36
DBP (mmHg) (N = 414)	77.5 (8.8)	77.0 (6.2)	-0.5 (9.0)	t -1.04, p = 0.30

The mean UACR level decreased significantly by 142.9 mg/g over 12 months (Table 4). Overall, 71.8% of patients achieved at least a 30% reduction in UACR.

**Table 4:** Clinical characteristics of UACR and eGFR: difference from baseline to 12 months UACR: urine albumin-creatinine ratio, eGFR: estimated glomerular filtration rate.

Clinical characteristics (N = 433)	Baseline, mean (SD)	12 months, mean (SD)	Difference, mean (SD)	Test statistic, p-value
UACR (mg/g)	207.8 (426.8)	64.9 (134.1)	-142.9 (18.2)	t -7.71, p < 0.001
eGFR (mL/min/1.73 m^2^)	82.0 (23.1)	84.0 (18.5)	+1.9 (0.9)	t 2.00, p < 0.05

Changes in UACR categories over 12 months of treatment are shown in Table 5. The results indicate an increase in the proportion of patients in the A1 category, with corresponding decreases in the proportions of patients in the A2 and A3 categories from baseline to 12 months. At 12 months, the odds of patients being in the A1 category were 2.2 times higher than at baseline (p < 0.001).

**Table 5:** Proportion of patients across UACR categories at baseline and at 12 months UACR: urine albumin-creatinine ratio.

UACR category (N = 433)	At baseline	At 12 months
A1 (<30 mg/g), n (%)	90 (20.8)	158 (36.5)
A2 (30-299 mg/g), n (%)	271 (62.6)	233 (53.8)
A3 (≥300 mg/g), n (%)	72 (16.7)	42 (9.7)

**Figure 2:**
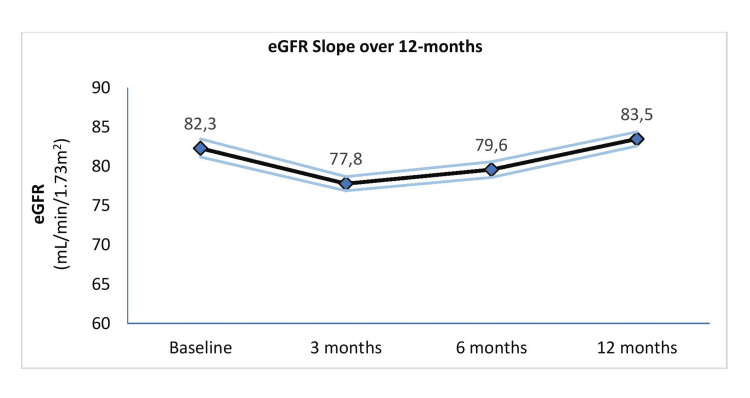
eGFR slope over the study duration (N = 399) The dark line represents the mean eGFR level, and the light lines represent 95% confidence interval for the eGFR slope.

In the overall study population, mean eGFR increased by 1.9 mL/min/1.73 m² from baseline to 12 months. Among the 399 patients with eGFR values available at all time points (baseline, 3 months, 6 months, and 12 months), an eGFR slope analysis was performed (Figure 2). Mean (95% CI) eGFR levels were 82.3 (81.2-83.5) mL/min/1.73 m² at baseline, 77.8 (76.9-78.7) mL/min/1.73 m² at 3 months, 79.6 (78.6-80.6) mL/min/1.73 m² at 6 months, and 83.5 (82.6-84.4) mL/min/1.73 m² at 12 months (F = 15.30, p < 0.001). Mean eGFR at 3 and 6 months was significantly lower than baseline, while the mean eGFR at 12 months was significantly higher than at 6 months.

## Discussion

This real-world study included 433 T2DM patients who were SGLT2-i-naïve, previously uncontrolled on a DPP4-i-based regimen, and initiated on the innovator FDC of empagliflozin and linagliptin, with follow-up in an outpatient setting for at least 12 months. Of these patients, 63.3% were male, 54.1% had HTN, 27.6% had a history of HF, and 10.3% had established ASCVD. At baseline, nearly 80% of patients had increased albuminuria levels, with the majority in the moderately increased albuminuria (A2) category. Over 12 months, combination therapy was associated with a significant HbA1c reduction of 1.5% (Table 2) and a mean body weight reduction of 3.3 kg (Table 3).

Mean eGFR increased slightly by 1.9 mL/min/1.73 m² over 12 months. In the subset of 399 patients with creatinine values recorded at baseline, 3 months, and 12 months, the eGFR slope analysis showed an initial mean decline of 4.5 mL/min/1.73 m² at 3 months, followed by an upward trajectory, ultimately reaching 1.9 mL/min/1.73 m² above baseline. Mean UACR decreased significantly by 142.9 mg/g, from 207.8 mg/g at baseline to 64.9 mg/g at 12 months. Overall, 71.8% of patients achieved at least a 30% reduction in UACR from baseline (Table 4), with 2.2-fold greater odds of being in the A1 category at 12 months.

Our real-world study observed significant improvements in glycemic parameters and body weight, consistent with the known clinical effects of this combination [11]. The majority of patients achieved clinically meaningful reductions in albuminuria and stabilization of eGFR levels. Across the study population, there was a modest increase in mean eGFR of 1.9 mL/min/1.73 m² over 12 months, with more pronounced improvement in older patients, had baseline HbA1c < 7%, or had UACR in the A3 category.

The eGFR-slope analysis, performed in a subset of 399 patients, demonstrated the characteristic initial dip in eGFR associated with SGLT2-i therapy [6,7]. Specifically, there was a mean decline of 4.5 mL/min/1.73 m² at three months, corresponding to an average initial dip of 5.5% in eGFR. Following this initial decline, eGFR levels significantly recovered by 12 months. Slowing the rate of eGFR decline is a key therapeutic goal for patients with T2DM and CKD [6-8], as evidence suggests that a faster eGFR decline may increase the long-term risk of major macrovascular and renal events by 30% [20]. Moreover, slowing eGFR decline by 0.69 mL/min/1.73 m² per year is associated with a meaningful reduction in the risk of progressing to end-stage kidney disease [21]. Although our study did not include a control group, the descriptive analysis suggests that the empagliflozin-linagliptin FDC may contribute to long-term stabilization of eGFR levels.

To assess individual patient-level variations, an exploratory analysis was conducted using 324 case records with a second confirmatory eGFR measurement within the first three months of treatment. Among these, 126 patients (38.9%) did not experience an eGFR dip in the first three months. A post hoc analysis of the EMPA-REG OUTCOME study reported a similar non-dipping pattern in 30.5% of participants [22], and this status did not affect the cardiorenal benefits of empagliflozin. The analysis also identified six patients (1.9%) who experienced an eGFR dip of >30% within the first four weeks of treatment. While SGLT2-i therapy may occasionally lead to acute renal impairment in patients predisposed to volume depletion [6,7], none of these patients met the criteria for acute kidney injury according to the RIFLE classification (>50% acute decline in eGFR) [23]. Current KDIGO guidelines advise that in patients with CKD who initiate hemodynamically active therapies, eGFR reductions of >30% on subsequent testing exceed expected variability and warrant evaluation. However, clinicians are cautioned against prematurely discontinuing these kidney-protective agents, as early eGFR dips are typically reversible and do not indicate drug toxicity [7].

In the study population, a modest increase in mean eGFR of 1.9 mL/min/1.73 m² was observed from baseline to 12 months. While this may partly reflect physiological variability in serum creatinine, an SGLT2-i-mediated correction of abnormally suppressed kidney function is also plausible in patients with intact nephrons. In early CKD, hemodynamic congestion or diuretic-induced volume depletion can compromise renal perfusion and reduce eGFR [24,25]. Treatment with an SGLT2i could favorably influence renal hemodynamics by decongesting volume overload or reducing the need for diuretics, potentially improving renal blood flow. Additionally, anemia of chronic disease can physiologically impair renal perfusion [26]. SGLT2-i-mediated improvements in iron metabolism [27] and cardiovascular function may further support enhanced renal circulation, although the relationship between hemoglobin changes and eGFR was not evaluated in this study. Finally, improvements in myocardial function among patients with ASCVD [28] may also contribute to better renal perfusion and eGFR correction. These proposed mechanisms for the modest eGFR increase observed in this study remain hypotheses and warrant further experimental investigation.

In addition, the reduction in mean UACR levels demonstrated statistical significance, likely attributable to both agents in the FDC, as supported by prior studies of each drug individually. In this study, mean UACR decreased from 207.8 mg/g at baseline to 64.9 mg/g at 12 months, representing a mean reduction of 142.9 mg/g. Notably, 71.8% of patients achieved at least a 30% reduction in UACR levels from baseline, and the 12-month treatment was associated with a 2.2-fold increase in the odds of patients being in the A1 UACR category compared to baseline (Table 5). At baseline, nearly 80% of patients had elevated albuminuria, with most in the moderately increased category. These findings highlight the reversible nature of albuminuria, particularly when detected early and managed with evidence-based therapy. Importantly, severely increased albuminuria (UACR ≥ 300 mg/g) is associated with a significantly higher risk of cardiorenal events and mortality [6]. Our study observed meaningful improvements in achieving normo-albuminuria among patients with T2DM and elevated albuminuria over 12 months of therapy.

Previous evidence suggests that achieving at least a 30% reduction in UACR within the first six months of treatment may translate into long-term clinical benefits for CKD outcomes [29]. A key observation from this study is that clinically meaningful UACR reductions were consistently seen across demographic and clinical subgroups. However, since this study reflects outcomes in a tertiary-care setting over a 12-month period, these findings may not be directly generalizable to primary or secondary care contexts.

In this study, baseline comorbidities included HTN in over half of the population, HF in more than one-fourth, and ASCVD in over one-tenth of patients. Notably, the primary kidney outcomes were consistently observed with the use of empagliflozin-linagliptin FDC regardless of the presence of these comorbidities (data not shown), supporting the notion that the renal benefits of this treatment regimen may be independent of its cardio-metabolic effects.

Reductions in glycemic parameters and body weight observed in this study were consistent with the known clinical effects of the combination. While the overall study population showed nominal but statistically non-significant reductions in SBP and DBP, patients with elevated baseline blood pressure experienced statistically significant reductions.

The results of this study align with existing real-world evidence on this combination for clinical parameters. In a study of 251 patients, linagliptin and empagliflozin demonstrated favorable effects on glycemic control, along with a well-tolerated safety profile [30]. Another observational study of 498 patients assessed effectiveness over 12 weeks, showing mean changes in HbA1c, FPG, SBP, DBP, eGFR, and body weight of -1.1%, -45 mg/dL, -12 mmHg, -5 mmHg, +5 mL/min/1.73 m², and -5 kg, respectively [31]. A separate retrospective analysis of 347 patients reported stabilization of renal function, particularly from the seventh week of treatment [32]. In North India, a real-world study of 50 T2DM patients with CKD treated with this FDC showed a reduction in UACR from 547 mg/g at baseline to 176 mg/g at 12 months, with regression of macroalbuminuria in 63% of patients [33].

This study has limitations inherent to its observational, retrospective design and the absence of a control group. Results represent two tertiary-care clinics in eastern India and may be influenced by confounding factors such as lifestyle modification, antihypertensive dose titration, and inherent variability in individual eGFR and UACR assessments. Nevertheless, the substantial sample size, 12-month follow-up, and detailed case record documentation strengthen the value of this real-world evidence. The findings also generate hypotheses for further scientific exploration.

## Conclusions

This real-world evidence suggests that in patients with T2DM who were previously uncontrolled on a DPP4-i-based antihyperglycemic regimen and were SGLT2-i-naïve, use of the original FDC of empagliflozin and linagliptin may be associated with meaningful reductions in UACR levels and stabilization of the eGFR slope over 12 months. These effects were observed consistently, regardless of underlying cardiometabolic risk status. The changes in glycemia, body weight, and blood pressure were also in line with the known clinical effects of this combination. As this is a descriptive analysis with potential for confounding, the findings should be interpreted cautiously and viewed as hypothesis-generating. Still, the observed improvements in eGFR trajectories and albuminuria reduction in patients with T2DM and early CKD provide a basis for further scientific investigation.

## References

[1] Yajnik CS (2018). Confessions of a thin-fat Indian. Eur J Clin Nutr.

[2] Rani A, Satyavani K, Viswanathan V (2017). Stratifying renal risk and retinal involvement in South Indian type 2 diabetic patients: using the KDIGO classification. Int J DiabetolVasc Dis Res.

[3] Singh K, Kondal D, Jagannathan R (2024). Rate and risk factors of kidney function decline among South Asians with type 2 diabetes: analysis of the CARRS Trial. BMJ Open Diabetes Res Care.

[4] Zoungas S, Arima H, Gerstein HC (2017). Effects of intensive glucose control on microvascular outcomes in patients with type 2 diabetes: a meta-analysis of individual participant data from randomised controlled trials. The Lancet Diabetes and Endocrinology.

[5] Samson SL, Vellanki P, Blonde L (2023). American Association of Clinical Endocrinology Consensus Statement: comprehensive type 2 diabetes management algorithm - 2023 Update. Endocr Pract.

[6] Kidney Disease: Improving Global Outcomes (KDIGO) CKD Work Group (2024). KDIGO 2024 Clinical Practice Guideline for the Evaluation and Management of Chronic Kidney Disease. Kidney Int.

[7] de Boer IH, Khunti K, Sadusky T (2022). Diabetes management in chronic kidney disease: a consensus report by the American Diabetes Association (ADA) and Kidney Disease: Improving Global Outcomes (KDIGO). Diabetes Care.

[8] Writing Group for the CKD Prognosis Consortium (2023). Estimated glomerular filtration rate, albuminuria, and adverse outcomes. An individual-participant data meta-analysis. JAMA.

[9] American Diabetes Association Professional Practice Committee (2025). Summary of revisions: standards of care in diabetes-2025. Diabetes Care.

[10] Del Prato S, Felton AM, Munro N, Nesto R, Zimmet P, Zinman B (2007). Improving glucose management: ten steps to get more patients with type 2 diabetes to glycaemic goal. Recommendations from the Global Partnership for Effective Diabetes Management. Int J Clin Pract Suppl.

[11] (2025). Boehringer Ingelheim. Glyxambi prescribing information. https://pro.boehringer-ingelheim.com/in/products/glyxambi/prescribing-information.

[12] Fadini GP, Bonora BM, Mayur S, Rigato M, Avogaro A (2018). Dipeptidyl peptidase-4 inhibitors moderate the risk of genitourinary tract infections associated with sodium-glucose co-transporter-2 inhibitors. Diabetes Obes Metab.

[13] Wanner C, Inzucchi SE, Lachin JM (2016). Empagliflozin and progression of kidney disease in type 2 diabetes. N Engl J Med.

[14] Hadjadj S, Cooper ME, Steubl D (2024). Empagliflozin and rapid kidney function decline incidence in type 2 diabetes: an exploratory analysis from the EMPA-REG OUTCOME trial. Kidney Med.

[15] The Nuffield Department of Population Health Renal Studies Group, The SGLT2 Inhibitor Meta-Analysis Cardio-Renal Trialists' Consortium (2022). Impact of diabetes on the effects of sodium glucose co-transporter-2 inhibitors on kidney outcomes: collaborative meta-analysis of large placebo-controlled trials. Lancet.

[16] McGuire DK, Shih WJ, Cosentino F (2021). Association of SGLT2 inhibitors with cardiovascular and kidney outcomes in patients with type 2 diabetes. A meta-analysis. JAMA Cardiol.

[17] Graefe-Mody U, Retlich S, Friedrich C (2012). Clinical pharmacokinetics and pharmacodynamics of linagliptin. Clin Pharmacokinet.

[18] Rosenstock J, Perkovic V, Johansen OE (2019). Effect of linagliptin vs placebo on major cardiovascular events in adults with type 2 diabetes and high cardiovascular and renal risk. The CARMELINA randomized clinical trial. JAMA.

[19] Perkovic V, Toto R, Cooper ME (2020). Effects of linagliptin on cardiovascular and kidney outcomes in people with normal and reduced kidney function: secondary analysis of the CARMELINA randomized trial. Diabetes Care.

[20] Inker LA, Collier W, Greene T (2023). A meta-analysis of GFR slope as a surrogate endpoint for kidney failure. Nat Med.

[21] Kraus BJ, Weir MR, Bakris GL (2021). Characterization and implications of the initial estimated glomerular filtration rate 'dip' upon sodium-glucose cotransporter-2 inhibition with empagliflozin in the EMPA-REG OUTCOME trial. Kidney Int.

[22] Ricci Z, Cruz D, Ronco C (2008). The RIFLE criteria and mortality in acute kidney injury: a systematic review. Kidney Int.

[23] Tamayo-Gutierrez A, Ibrahim HN (2022). The kidney in heart failure: the role of venous congestion. Methodist Debakey Cardiovasc J.

[24] Shah PB, Soundararajan P, Sathiyasekaran BW, Hegde SC (2017). Diuretics for people with chronic kidney disease. Cochrane Database Syst Rev.

[25] Saraf SL, Hsu JY, Ricardo AC (2020). Anemia and incident end-stage kidney disease. Kidney360.

[26] Packer M (2023). Alleviation of functional iron deficiency by SGLT2 inhibition in patients with type 2 diabetes. Diabetes Obes Metab.

[27] Theofilis P, Antonopoulos AS, Katsimichas T (2022). The impact of SGLT2 inhibition on imaging markers of cardiac function: a systematic review and meta-analysis. Pharmacol Res.

[28] Heerspink HJL, Greene T, Tighiouart H (2019). Change in albuminuria as a surrogate endpoint for progression of kidney disease: a meta-analysis of treatment effects in randomised clinical trials. Lancet Diabetes Endocrinol.

[29] Oshima M, Jun M, Ohkuma T (2019). The relationship between eGFR slope and subsequent risk of vascular outcomes and all-cause mortality in type 2 diabetes: the ADVANCE-ON study. Diabetologia.

[30] Kovil R, Saboo B, Shah K, Padhye D, Chudasama D, Raj V, Shaikh N (2020). Single-pill combination of empagliflozin and linagliptin in real world Indian type 2 diabetes patient (GRID). J Assoc Physicians India.

[31] Gupta A, Khalse M, Bhargava A (2020). 2206-PUB: efficacy of fixed-dose combination of empagliflozin and linagliptin in addition to the ongoing treatment in type 2 diabetes: real-world observational study. Diabetes.

[32] Gupta A, Malhotra P, Jamwal V, Khalse M (2021). A retrospective analysis of fixed combination of empagliflozin and linagliptin in addition to the existing treatment for its clinical effectiveness in adults with type 2 diabetes: a real-world clinical experience. J Assoc Physicians India.

[33] Mahajan S (2024). Effectiveness and safety of a single-pill combination of empagliflozin/linagliptin in individuals with uncontrolled type 2 diabetes with CKD: a single-centre outpatient clinic real-world experience. CJD.

